# Evaluation of pesticide residues in commercial Swiss beeswax collected in 2019 using ultra-high performance liquid chromatographic analysis

**DOI:** 10.1007/s11356-021-18363-9

**Published:** 2022-01-11

**Authors:** Joshua N. G. Marti, Verena Kilchenmann, Christina Kast

**Affiliations:** Agroscope, Swiss Bee Research Centre, Schwarzenburgstrasse 161, 3003 Bern, Switzerland

**Keywords:** *Apis mellifera*, Beeswax, *Varroa destructor*, Acaricide, Pesticide, Residue

## Abstract

The aim of this study was to determine residue levels of pesticides in Swiss commercial beeswax. Foundation samples were collected in 2019 from nine commercial manufacturers for analysis of 21 pesticides using ultra-high performance liquid chromatography. Individual samples showed the variability and residue ranges and pooled samples represented the average annual residue values of the Swiss production. In total, 17 pesticides were identified and 13 pesticides were quantified. They included 13 acaricides and/or insecticides, two fungicides as well as a synergist and a repellent. The means calculated from individual samples were similar to the average annual residue values for most tested pesticides. Mean values of 401, 236, 106 and 3 μg·kg^−1^ were obtained for the beekeeping-associated contaminants coumaphos, *tau*-fluvalinate, bromopropylate and N-(2,4-Dimethylphenyl)-formamide (DMF; breakdown product of amitraz), respectively. For the other pesticides, the mean values were 203 μg·kg^−1^ (synergist piperonyl butoxide), 120 μg·kg^−1^ (repellent *N*,*N*-Diethyl-3-methylbenzamide, DEET), 19 μg·kg^−1^ (chlorfenvinphos) and 4 μg·kg^−1^ ((E)-fenpyroximate), while the means for acrinathrin, azoxystrobin, bendiocarb, boscalid, chlorpyrifos, flumethrin, permethrin, propoxur and thiacloprid were below the limit of quantification (< *LOQ*). Individual samples contained from seven to 14 pesticides. The ranges of values for coumaphos and piperonyl butoxide (from 14 to 4270 μg·kg^−1^; from 6 to 1555 μg·kg^−1^, respectively) were larger as compared to the ranges of values for DEET and *tau*-fluvalinate (from < *LOQ* to 585 μg·kg^−1^; from 16 to 572 μg·kg^−1^, respectively). In conclusion, the most prominent contaminants were the pesticides coumaphos and *tau*-fluvalinate, which are both acaricides with previous authorization for beekeeping in Switzerland, followed by piperonyl butoxide, a synergist to enhance the effect of insecticides.

**Graphical abstract:**

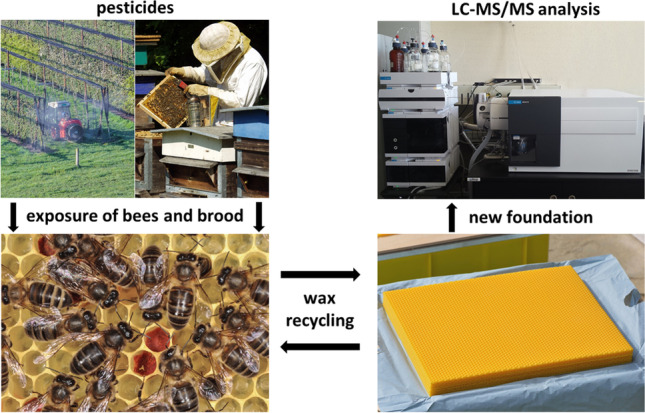

## Introduction

Honeybees (*Apis mellifera*) play an important role as pollinators in ecosystems and agriculture. However, many studies report colonies losses due to various reasons, including beekeeping practice, honey bee diseases, especially the mite *Varroa destructor* and its associated viruses, and exposure to pesticides (Rosenkranz et al. 2010; Steinhauer et al. 2018). When beekeepers use veterinary drugs to treat mite infestation, these substances can accumulate in the beehive. In beeswax, lipophilic acaricides are the most frequently detected pesticides at the highest levels (Bogdanov 2004; Boi et al. 2016; Calatayud-Vernich et al. 2017; Chauzat et al. 2011; El Agrebi et al. 2020; Fulton et al. 2019; Wallner 1999) probably related to the fact that they are directly applied into the hive. Plant-protecting products used in agriculture are an additional source of pesticide residues in beeswax. Bees may bring these pesticides into the colonies when they forage for pollen and nectar. Thus, honey bees can be exposed to a variety of pesticides. Hydrophilic substances with a low logarithmic octanol–water partition coefficient tend to accumulate in honey, while lipophilic pesticides with a high logarithmic octanol–water partition coefficient preferentially accumulate in beeswax (Murcia Morales et al. 2020). The accumulation of pesticides in beeswax is especially critical, since the highly sensitive honeybee larvae are exposed to these pesticides through direct contact to the beeswax or indirectly if the pesticides migrate from the beeswax into the larval jelly (Kast and Kilchenmann 2022; Wilmart et al. 2021).

In good beekeeping practice, the brood combs are replaced after three to four years. The old comb wax is recycled by melting the combs together with capping wax in water vapour producing blocks for the manufacturing of new foundation sheets. Beekeepers place frames containing these new foundations in the hives for the bees to construct new combs. Many pesticides remain in the recycled wax and are still present in the newly produced wax foundation sheets (Bogdanov et al. 1998; Martel et al. 2007). Even years after a product is no longer used, residues of the active ingredients can be measured in beeswax (Kast et al. 2021), thus constantly exposing the bees to pesticides and possibly affecting honeybee health (Kast and Kilchenmann 2022; Wu et al. 2011).

Recent studies reported multiple pesticide residues in European beeswax (El Agrebi et al. 2020; Martinello et al. 2020; Perugini et al. 2018; Shimshoni et al. 2019). In Switzerland, the three beekeeping-associated acaricides coumaphos, *tau-*fluvalinate and bromopropylate have been monitored for three decades using gas chromatography (Kast et al. 2021). So far, there is no recent data on additional pesticide residues, including pesticides related to agricultural use. Therefore, an alternative method based on liquid chromatography was validated with the aim to compare residue levels in Swiss beeswax to the levels in beeswax of other countries.

First, 98 individual foundation samples were analysed to obtain the prevalence of a given pesticide alongside the range. Analysis of individual batches/samples gives a good idea of the variability and the maximal residue levels that customers can expect when buying foundation sheets. In a second step, samples were pooled according to the weight of each production batch, which led to results that came as close as possible to an overall average value for the annual production of Switzerland. These data can serve as a baseline for comparison with future residue levels.

## Material and methods

### Material

Caffeine (Art. C0750), acrinathrin (PESTANAL, Art. 46415), coumaphos (PESTANAL, Art. 45403), chlorpyrifos (PESTANAL, Art. 45395), flupyradifurone (PESTANAL, Art. 37050) and thiacloprid (PESTANAL, Art. 37905) were purchased from Sigma-Aldrich (Seelze, Germany). Azoxystrobin (Art. C10413000), bendiocarb (Art. C10460000), boscalid (Art. C10663000), bromopropylate (Art. C10762000), chlorfenvinphos (Art. C11290000), *N*,*N*-Diethyl-3-methylbenzamide (DEET, Art. C12100000), deltamethrin (Art. C12120000), *N*-(2,4-Dimethylphenyl)-formamide (DMF, Art. C12737000), fenitrothion (Art. C13480000), (E)-fenpyroximate (Art. C13545000), flumethrin (Art. C13719000), *tau*-fluvaliante (Art. C13870000), permethrin (Art. C15990000), piperonyl butoxide (Art. C16240000), propoxur (Art. C16500000) and zeta-cypermethrin (Art. C11890500) were obtained from Dr Ehrenstorfer (Augsburg, Germany). Acetonitrile of SupraSolv quality and 2-propanol of LiChrosolv quality were purchased from Merck (Darmstadt, Germany). Formic acid solution 50% (Art. 09676) was obtained from Honeywell Fluka (Buchs, Switzerland). Ammonium formate (Art. 70221) was purchased from Supelco (Darmstadt, Germany). Bondesil PSA 40 μm (Art. 12213024) and Bondesil C18 40 μm (Art. 12213012) were obtained from Agilent Technologies (USA). Syringe filters 0.45 μm polyamide (Art. 729049) were purchased from Machery-Nagel (Düren, Germany). The water used for the mobile phases was purified with a Milli-Q IQ 7000 system.

### Beeswax for blank wax extracts

Before the study was started, analysis was performed on wax blocks from three organic apiaries to find a suitable beeswax that can be used as blank wax extract. The beeswax that was chosen was from newly constructed combs produced in the year 2012, since it contained the lowest overall level of pesticide residues. Nevertheless, it contained DEET at approximately 20 μg·kg^−1^ and azoxystrobin at approximately 7 μg·kg^−1^.

### Beeswax samples from the manufacturers of foundations

This study on pesticide residues in commercial beeswax included all major commercial manufacturers in Switzerland. Each of the nine participating manufacturers produced between 400 and 28 000 kg of foundations sheets during the year 2019. Wax samples were collected all year long from each produced batch and stored in the dark at − 20 °C. At the beginning of the year 2020, all the manufacturers sent their samples (321 samples in total) to the Swiss Bee Research Centre together with the information on the size of each productions batch. The number of samples/batches per producer ranged from 3 to 171. For single batch analysis, approximately every third batch was sampled (for each manufacturer: 43 out of 171; 13 out of 49; 8 out of 31; 7 out of 27; 6 out of 21; 8 out of 8; 7 out of 7; 4 out of 4; 2 out of 3 samples), which led to an even distribution across the year and was more or less in proportion to the production volume of each manufacturer. In total, 98 out of 321 samples (31% of all samples) were analysed individually. In addition, representative samples were prepared for each manufacturer by combining wax from each sample/batch in proportion to the corresponding size of the production batch (Kast et al. 2021). Pooled samples have been previously analysed by gas chromatography (Kast et al. 2021). The pooled samples of the year 2019 were reanalysed in this study using the newly validated method based on liquid chromatography to obtain the annual residue values of 18 additional pesticides. From the annual residue values of the nine manufacturers, the average annual value of the residues for all Switzerland (later named annual value) was calculated. To obtain an annual value as close as possible to a true average value for Switzerland, the different amounts of foundations produced by each manufacturer during the year were taken into account. Values < *LOQ* were replaced with zero for the calculation.

### Sample preparation

The extraction procedure for all the tested pesticides was performed according to a modified QuEChERS (quick, easy, cheap, efficient, rugged, safe) method, which is based on a procedure previously described by Kast et al. (2020). Wax (0.48–0.52 g) was weighed into a 50 mL centrifugation tube and the exact mass was noted for later correction of the initial weight. Acetonitrile (5 mL) containing caffeine at 50 μg/L were added. The sample was placed in a hot water bath at 80 °C for 30 min to melt the wax. Subsequently, the sample was shaken vigorously by hand for 30 s and placed back in the hot water bath for another 10 min. This step was repeated three more times before the sample was placed at 80 °C for a final 10 min. Then the sample was allowed to cool to room temperature. Subsequently, the sample was placed at − 20 °C overnight, followed by centrifugation at 10000 g at 4 °C for 20 min on the next day. After the centrifugation step, 1 mL of the supernatant was pipetted into a 1.5 mL Eppendorf tube containing 25 mg PSA and 25 mg C18. The tube was mixed twice on the vortex stirrer for 30 s. Afterwards, the tube was stored in the freezer at − 20 °C overnight. The sample was then separated by centrifugation at 18000 g at 4 °C for 20 min. Finally, the supernatant was filtered through a 0.45 μm polyamide membrane into an auto sampler vial.

### UHPLC-MS/MS analysis

Three individual methods (A, B and C) were established for the analysis of the 21 pesticides with variable eluent gradients and ion source conditions of the mass spectrometer (MS). Method A allowed analysis of fenitrothion, chlorpyrifos, zeta-cypermethrin, deltamethrin, acrinathrin, permethrin and flumethrin. Method B was used for the analysis of DMF (the principal breakdown product of amitraz in wax foundations; Calatayud-Vernich et al. 2017), coumaphos, bromopropylate and *tau*-fluvalinate, and method C for flupyradifurone, thiacloprid, propoxur, bendiocarb, DEET, azoxystrobin, boscalid, chlorfenvinphos, piperonyl butoxide and (E)-fenpyroximate.

Ultra-high performance liquid chromatography (UHPLC) analysis was performed on an Agilent 1290 Infinity II system equipped with an auto sampler and coupled to an Agilent 6495C mass spectrometer (MS). For separation, a C18 reversed phase column (Acquity UPLC HSS T3 column, 100 Å, 1.8 μm, 2.1 mm × 100 mm; Waters) was used. The column temperature was 40 °C. The mobile phase A was 95% water + 5% acetonitrile + 0.01% formic acid + 5 mM ammonium formate and the mobile phase B was 5% water + 95% acetonitrile + 0.01% formic acid + 5 mM ammonium formate. Three individual gradients for methods A, B and C were used for the separation of the pesticides. The conditions of the gradients are listed in Table 1. The flow rate was 0.5 ml per min, and 2-propanol, acetonitrile and 0.01% formic acid in water were used to wash the needle. The temperature of the autosampler was 10 °C and the injection volume was 1 μL. An Agilent 6495C series tandem quadrupole MS system equipped with an electrospray ionisation source was used for detection of the various pesticides using mass fragmentation. The ion source conditions using positive mode electrospray ionisation for methods A, B and C are listed in Table 2, and the ion transitions for the pesticides are listed in Table 3. For each compound, one transition was used for quantification and two additional transitions for identification.

**Table 1:** LC-gradients for methods A, B and C

	Method A			Method B			Method C		
Step	Time^a^	A^b^	B^c^	Time	A	B	Time	A	B
1	0.25 min	100%	0%	0.25 min	100%	0%	0.25 min	100%	0%
2	6.60 min	20%	80%	6.60 min	0%	100%	4.00 min	60%	40%
3	13.00 min	0%	100%	12.00 min	0%	100%	6.60 min	0%	100%
4	13.01 min	100%	0%	12.01 min	100%	0%	9.01 min	100%	0%

^a^Time indicates the time at which the indicated ratio of A/B is present

^b^Mobile phase A: 95% water + 5% acetonitrile + 0.01% formic acid + 5 mM ammonium formate

^c^Mobile phase B: 5% water + 95% acetonitrile + 0.01% formic acid + 5 mM ammonium formate

**Table 2:** Ion source conditions of methods A, Band C

Method	A	B	C
Gas temp (°C)	130	180	250
Gas flow (L/min)	20	20	20
Nebulizer (psi)	30	30	60
SheathGasHeater (°C)	120	150	320
SheathGasFlow (L/min)	10	6	10
Capillary (V)	6000	6000	6000
VCharging	2000	2000	2000

**Table 3:** Ion transitions used for quantification and qualification

		Quantifier		Qualifier 1		Qualifier 2	
Analyte	Precursor ion (m/z)^a^	Product ion (m/z)	CE^b^ (V)	Product ion (m/z)	CE (V)	Product ion (m/z)	CE (V)
Acrinathrin	559.2	208.2	14	181.2	38	83.2	18
Azoxystrobin	404.1	372.3	14	344.3	26	329.3	34
Bendiocarb	244.1	167.2	6	109.1	18	81.2	42
Boscalid	343.0	271.3	38	272.3	34	140.1	18
Bromopropylate	444.0	208.9	42	408.7	6	152.9	66
Caffeine (IS)	195.1	138.2	18	110.1	26	42.3	74
Chlorfenvinphos	359.0	155.1	10	205.1	22	170.1	50
Chlorpyrifos	349.9	198.0	18	125.1	18	97.1	34
Coumaphos	363.0	226.8	30	306.7	18	210.8	34
Zeta-cypermethrin	433.1	191.1	14	416.3	6	127.1	34
DEET	192.1	119.1	18	91.2	34	65.2	58
Deltamethrin	521.0	279.1	14	504.1	6	172.1	34
DMF	150.1	106.9	22	106.0	38	77.0	50
Fenitrothion	278.0	125.1	22	246.2	18	109.1	18
(E)-fenpyroximate	422.2	366.4	18	138.1	34	135.1	34
Flumethrin	527.1	266.8	14	509.9	6	238.8	22
Flupyradifurone	289.1	126.1	22	90.1	50	73.1	80
*Tau*-fluvalinate	503.1	207.9	10	180.9	38	151.9	80
Permethrin	408.1	183.2	6	355.3	6	165.1	54
Piperonyl butoxide	356.2	177.2	14	119.2	42	91.2	62
Propoxur	210.1	111.1	14	168.2	2	93.1	26
Thiacloprid	253.0	126.1	22	90.2	46	73.1	78

^a^Mass/charge ratio of the ion

^b^Collision energy (volt)

Quantification was achieved through matrix-matched external calibration, using eight concentrations ranging from 0.05 μg·L^−1^ to 1000 μg·L^−1^. Concentrations of the samples were calculated based on the linear regression of the calibration samples and were performed with the software Agilent MassHunter.

Caffeine served as an internal standard. It was used for visual evaluation of extraction and injection, but no correctional factor was calculated. The limit of detection (*LOD*) was experimentally determined using spiked blank extracts. The *LODs* (signal to noise 10) for the various pesticides were between 0.04 and 10 μg·L^−1^ corresponding to values between 0.4 and 100 μg·kg^−1^ in wax (Table 4). Since our blank beeswax contained DEET and azoxystrobin, we could not experimentally determine the *LODs* for these two pesticides, hence the indication non-applicable (n.a.). The recoveries for the pesticides were tested at least at five spiking levels with at least five repetitions each. Recoveries of 80–120% were accepted. The final validated range for all 21 pesticides can be found in Table 4. The limits of quantification (*LOQ*) were defined as the lowest validated spike level, where the recoveries were above 80%. The *LOQs* for the tested pesticides ranged between 0.5 and 200 μg·kg^−1^ in wax (Table 4). We set the *LOQ* at levels above the blank values for DEET (20 μg·kg^−1^) and for azoxystrobin (10 μg·kg^−1^) to ensure proper quantification.

**Table 4:** Parameters for method validation, prevalence of pesticides and their mean, median, minimal, maximal and annual values

Compound	Application^a^	log P^b^	*LOD*^c^ (µg·kg^−1^)	*LOQ*^d^ (µg·kg^−1^)	Validated range (µg·kg^−1^)	Prevalence^e^ (%)	Mean^f^ (µg·kg^−1^)	Median (µg·kg^−1^)	Minimum (µg·kg^−1^)	Maximum (µg·kg^−1^)	Annual value^g^ (µg·kg^−1^)
Acrinathrin	a,i^1^	6.3^6^	20	100	100–10,000	2	< *LOQ*	< *LOQ*	< *LOD*	< *LOQ*	n.c.^i^
Azoxystrobin	f^1^	2.5^6^	n.a.^h^	10	10–1000	100	< *LOQ*	< *LOQ*	< *LOQ*	< *LOQ*	n.c.^i^
Bendiocarb	i^2^	1.7^2^	1	2.5	2.5–10,000	1	< *LOQ*	< *LOQ*	< *LOD*	< *LOQ*	n.c.^i^
Boscalid	f^1^	3.0^2^	2.5	5	5–5000	10	< *LOQ*	< *LOQ*	< *LOD*	9.9	n.c.^i^
Bromopropylate	a^1^	4.9^7^	50	50	50–10,000	97	106.4	105.4	< *LOQ*	207.7	69.7
Chlorfenvinphos	a,i^1^	3.8^6^	0.5	0.5	0.5–10,000	99	19.0	9.0	< *LOD*	185.1	23.1
Chlorpyrifos	i^1^	4.7^6^	1.6	8	8–10,000	99	< *LOQ*	< *LOQ*	< *LOD*	33.8	5.1
Coumaphos	a,i^1^	3.9^6^	1	1	1–10,000	100	400.9	294.7	14.2	4269.5	556.1
Zeta-cypermethrin	i^1^	6.6^2^	100	100	100–10,000	0	< *LOD*	< *LOD*	< *LOD*	< *LOD*	n.c.^i^
DEET	r, i^1^	2.0^8^	n.a.^h^	20	20–5000	100	119.9	70.1	< *LOQ*	585.4	107.4
Deltamethrin	i^1^	6.1^9^	100	200	200–10,000	0	< *LOD*	< *LOD*	< *LOD*	< *LOD*	n.c.^i^
DMF	a,i^1^	1.5^10^	1	1	1–10,000	49	2.9	< *LOQ*	< *LOD*	32.1	2.8
Fenitrothion	i^2^	3.3^2^	8	20	20–10,000	0	< *LOD*	< *LOD*	< *LOD*	< *LOD*	n.c.^i^
(E)-fenpyroximate	a^1^	5.7^2^	0.5	1	1–5000	92	4.4	1.3	< *LOD*	49.7	3.6
Flumethrin	a^3^	6.2^2^	25	40	40–10,000	51	< *LOQ*	< *LOQ*	< *LOD*	110.5	10.9
Flupyradifurone	i^4^	1.2^2^	1	2.5	2.5–10,000	0	< *LOD*	< *LOD*	< *LOD*	< *LOD*	n.c.^i^
*Tau*-fluvalinate	a,i^1^	7.0^6^	4	10	10–10,000	100	236.3	244.4	15.5	572.0	301.8
Permethrin	i^1^	6.1^6^	20	40	40–10,000	85	< *LOQ*	< *LOQ*	< *LOD*	250.3	54.0
Piperonyl butoxide	s^5^	4.8^2^	0.5	0.5	0.5–2000	100	202.5	93.5	6.1	1554.8	200.2
Propoxur	a,i^2^	0.1^2^	0.4	1	1–10,000	88	< *LOQ*	< *LOQ*	< *LOD*	9.2	0.6
Thiacloprid	i^1^	1.3^2^	0.5	1	1–10,000	2	< *LOQ*	< *LOQ*	< *LOD*	< *LOQ*	n.c.^i^

^a^a = acaricide, i = insecticide, f = fungicide, r = repellent, s = synergist

^b^Octanol-water partition coefficient and the respective literature source

^c^Limit of detection (*LOD*) of pesticide in wax

^d^Limit of quantification (*LOQ*) of pesticide in wax

^e^Percentage of individual samples containing a given pesticide in concentrations > *LOD*

^f^Calculated of individual samples, concentrations < *LOQ* were replaced with zero

^g^Average annual residue value for beeswax produced in Switzerland, concentrations < *LOQ* were replaced with zero for calculation

^h^Non-applicable (n.a.), since no pesticide free blank free of azoxystrobin and DEET available (see material and method)

^i^Not calculable (n.c.), the annual values of all manufacturers < *LOQ*

^1^El Agrebi et al. (2020)

^2^Lewis et al. (2016)

^3^El Agrebi et al. (2019)

^4^Guo et al. (2021)

^5^Tozzi (1999)

^6^Murcia Morales et al. (2020)

^7^Escuder-Gilabert et al. (2001)

^8^Jackson et al. (2008)

^9^Johnson et al. (2010)

^10^Anonymous (2021)

The mean and median values for pesticides were calculated across the 98 individual foundation samples, taking values below *LOQ* as 0. For some pesticides with only a few quantifiable measurements (boscalid, chlorpyrifos, DMF, flumethrin, permethrin and propoxur), a mean and/or median below *LOQ* was obtained.

## Results

In total, 17 out of 21 pesticides were detected in the analysed foundation samples, among which 13 pesticides could be quantified (Table 4). The values of mean calculated from the individual samples were similar to the annual residue values (Table 4). The absolute differences ranged from 0.1 μg·kg^−1^ (DMF) to 155 μg·kg^−1^ (coumaphos) (Table 4).

**Fig. 1:**
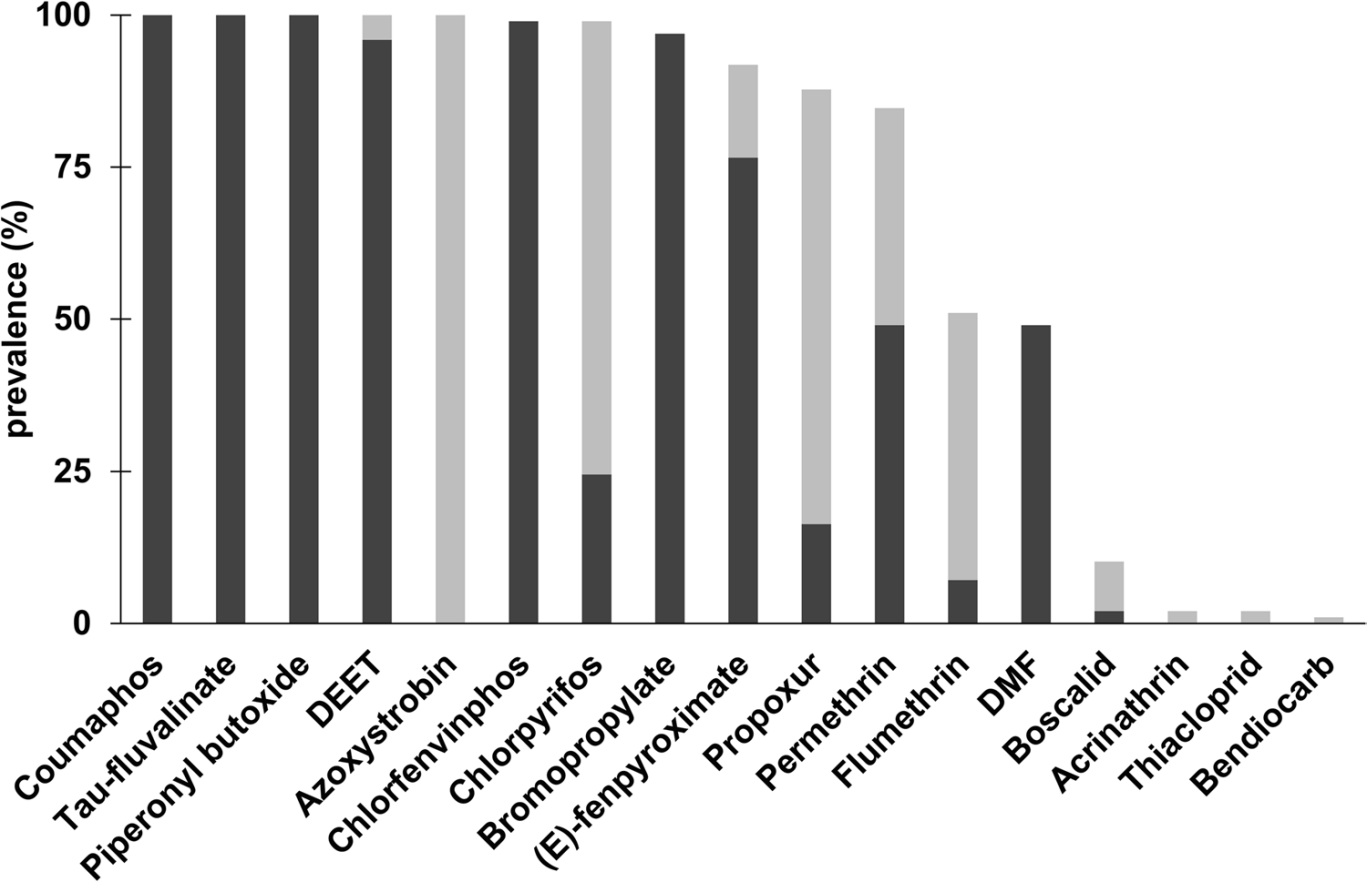
Prevalence of the 21 analysed pesticides in beeswax: The prevalence was calculated as percentage of detections above *LOD* compared to all 98 individual samples. Light grey bars show the detections *LOD* < value < *LOQ*, dark grey bars are detections > *LOQ*. The individual limits of detection (*LOD*) for each pesticide are listed in Table 4

On average, 12 different pesticides were found per sample, with a minimum of seven and a maximum of 14 pesticides per foundation sample. The prevalence of a given pesticide was calculated as the percentage of detections above the *LOD*. The values between the *LOD* and *LOQ* were included in this calculation, since information about the presence of the pesticide would otherwise be lost for pesticides with a high *LOQ* (e.g. Azoxystrobin and DEET were present in the blank wax and thus could not be quantified below 10 and 20 μg·kg^−1^). Out of the 21 analysed pesticides, 11 pesticides were found in nearly all the tested foundation samples (85–100%, Fig. 1). Among them were the beekeeping-associated acaricides coumaphos, *tau*-fluvalinate and bromopropylate, alongside the insecticides chlorfenvinphos, chlorpyrifos, propoxur and permethrin. Furthermore, the synergist piperonyl butoxide, the fungicide azoxystrobin (however below the *LOQ*), the acaricide (E)-fenpyroximate (agricultural use) as well as DEET, a repellent were detected in most samples (Fig. 1). The beekeeping-associated acaricides flumethrin and DMF were detected in approximately 50% of samples, while the fungicide boscalid was detected in 10% of the samples (Fig. 1). Additionally, a small number of samples contained one or several of the insecticides acrinathrin, thiacloprid or bendiocarb (Fig. 1). Zeta-cypermethrin, fenitrothion, flupyradifurone and deltamethrin were not detected in the tested foundation samples (Table 4).

The highest mean values were measured for coumaphos (400.9 μg·kg^−1^), *tau*-fluvalinate (236.3 μg·kg^−1^), piperonyl butoxide (202.5 μg·kg^−1^), DEET (119.9 μg·kg^−1^) and bromopropylate (106.4 μg·kg^−1^) (Table 4). For bromopropylate, *tau*-fluvalinate and DEET, the mean and median values were similar, while larger deviations between these values were observed for coumaphos and piperonyl butoxide due to some individual samples containing higher residue levels (Table 4). A mean value of 19.0, 4.4 and 2.9 μg·kg^−1^ was obtained for chlorfenvinphos, (E)-fenpyroxymate and DMF, respectively, while the mean values of nine pesticides, namely, acrinathrin, azoxystrobin, bendiocarb, boscalid, chlorpyrifos, flumethrin, permethrin, propoxur and thiacloprid were < *LOQ*.

**Fig. 2:**
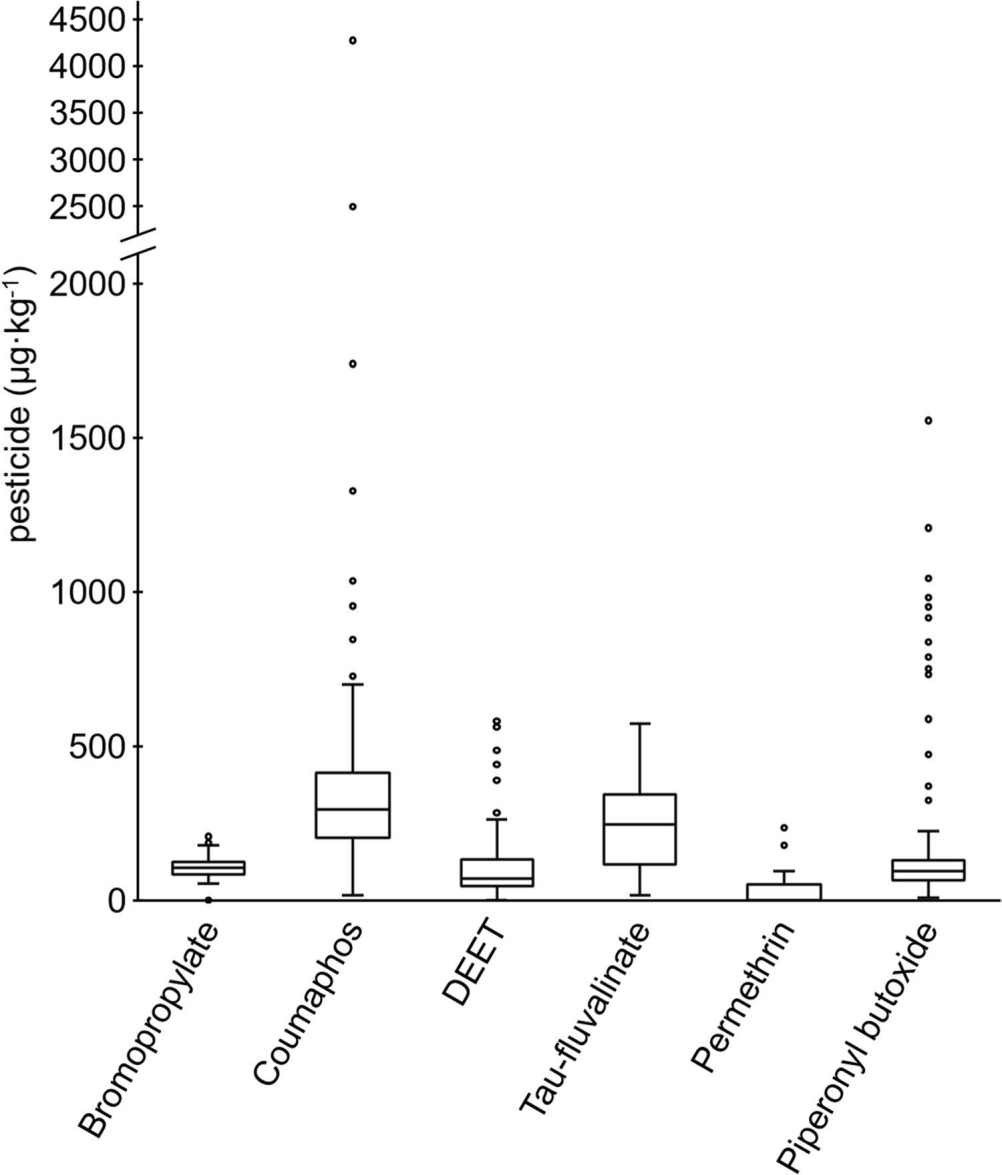
Box plots for pesticides with the six highest maximums: The line at the centre of each box indicates the median, while the edges of the boxes indicate the upper and lower quartiles. The whiskers contain a distance up to the highest value within 1.5 times the interquartile range. Residue values outside this range are indicated as circles

Residue levels of coumaphos and piperonyl butoxide showed a large variability, with residue levels from 14.2 to 4269.5 μg·kg^−1^ and from 6.1 to 1554.8 μg·kg^−1^, respectively, while residue levels regarding *tau*-fluvalinate, bromopropylate, DEET and permethrin were more uniformly distributed in the tested foundation samples (Fig. 2).

## Discussion

In total, 17 different pesticides were identified, of which 13 could be quantified. Among these pesticides, 11 were present in the majority of the tested production batches. The quantitatively most prominent contaminants were coumaphos and *tau*-fluvalinate, which are two acaricides with previous authorisation for beekeeping in Switzerland. The highest residue values in individual foundations were obtained for coumaphos, followed by piperonyl butoxide, a synergist frequently applied as part of some plant protection products.

The two different approaches, calculating the annual values from pooled samples and determining the means of individually analysed samples, produced comparable results for most pesticides. The sampling for analysis of individual foundations included nine manufacturers and was spread regularly over the whole year 2009, which was probably a prerequisite. Recently, analysing many individual samples has become more feasible because sample preparation and analytical methods have become less time-consuming.

The results of this study are in line with the numerous previous studies reporting that acaricides, especially coumaphos and *tau*-fluvalinate, are among the most frequently detected pesticides in beeswax (Bogdanov 2004; Calatayud-Vernich et al. 2017; Fulton et al. 2019; Lozano et al. 2019; Mullin et al. 2010; Perugini et al. 2018; Shimshoni et al. 2019; Spiewok 2017). A product containing coumaphos at a high dose (CheckMite +) has been authorised in Switzerland for beekeeping (Kast et al. 2020). However, it was rarely used by beekeepers in the last few years (Kast et al. 2021). Its authorisation in Switzerland expired in September 2021. A *tau*-fluvalinate-containing product was authorised in Switzerland for beekeeping until December 2006 and the residue levels of *tau*-fluvalinate have decreased since the 2000s (Kast et al. 2021). However, slightly increased values have been observed since 2015 (Kast et al. 2021), possibly due to an import of wax from countries where *tau*-fluvalinate is authorised for beekeeping or as a plant protection product (D’Ascenzi et al. 2019). Furthermore, bromopropylate is still present in 97% of samples up to a maximal level of 208 μg·kg^−1^, even though the authorisation period in Switzerland ended in 1999 (Kast et al. 2021). Just like *tau*-fluvalinate, residue levels have been slowly decreasing over time. The three acaricides coumaphos, *tau*-fluvalinate and bromopropylate have high octanol–water partition coefficients of 3.9, 7.0 and 4.9 (Escuder-Gilabert et al. 2001; Murcia Morales et al. 2020), respectively. Thus, they have lipophilic character, accumulate preferentially in wax and remain in recycled wax over many years. The use of the more hydrophilic organic acids for mite control instead of lipophilic acaricides can help to keep residue levels in beeswax low.

In this study, mean residue levels were 401 μg·kg^−1^ and 236 μg·kg^−1^ for coumaphos and *tau*-fluvalinate, respectively. These levels were comparable to the levels measured in the beeswax of neighbouring countries, such as Germany (means of 720 and 230 μg·kg^−1^, Shimshoni et al. 2019), France (means of 648 and 220 μg·kg^−1^, Chauzat et al. 2011) and Italy (in 2014, means of 217 and 130 μg·kg^−1^, Boi et al. 2016). Higher average levels of coumaphos and *tau*-fluvalinate were reported, for example, in a Spanish study (means of 9486 and 1085 μg·kg^−1^, Calatayud-Vernich et al. 2017) or in a North American study (means of 3300 and 7474 μg·kg^−1^, Mullin et al. 2010). We observed a high variability of coumaphos levels between the batches, with a maximal value of 4270 μg·kg^−1^, which is in the same order of magnitude to maximal values reported for France (4113 μg·kg^−1^, Chauzat et al. 2011) and Germany (10 900 μg·kg^−1^, Shimshoni et al. 2019). However, the individual batches were more homogeneous regarding *tau*-fluvalinate residue levels. The maximal value of 572 μg·kg^−1^ is comparable to a maximal level measured in French beeswax (446 μg·kg^−1^, Chauzat et al. 2011). In contrary, higher maximal residue levels were previously reported in German wax (8500 μg·kg^−1^, Spiewok 2017) although *tau*-fluvalinate has never been authorised for beekeeping in Germany (Wallner 2014).

Coumaphos levels of up to 20 000 μg kg^−1^ in wax were non-lethal to honey bee larvae, as previously shown in an in vitro model (Kast and Kilchenmann 2022), suggesting that the coumaphos levels currently measured in Swiss beeswax or in wax of the neighbouring countries most likely do not affect brood development. Furthermore, a recent study showed that wax foundations containing coumaphos, *tau*-fluvalinate and thymol at a concentration of 10 000 μg·kg^−1^ each did not increase brood mortality rates (Alkassab et al. 2020). Additional studies on lethal and sub-lethal effects of the various pesticides in beeswax as well as the synergistic effects of a mix of pesticides on honeybees would be helpful to determine maximal residue levels for beeswax.

Coumaphos, *tau*-fluvalinate and bromopropylate (three out of 21 pesticides described in the current study) have been previously analysed in a long-term survey for Swiss beeswax (Kast et al. 2021). Thus, the pooled samples of the year 2019 (but not the individual samples) have been analysed using two different analytical procedures. In the previous survey, the samples were purified on a florisil column and analysed by gas chromatography (Kast et al. 2021), while in the current study, the samples were extracted with modified QuEChERS procedure and analysed by liquid chromatography. Despite the different extraction procedures, the annual values (year 2019) obtained in the previous study for coumaphos (410 μg·kg^−1^), *tau*-fluvalinate (380 μg·kg^−1^) and bromopropylate (79 μg·kg^−1^) were comparable to the annual values obtained in this current survey (556, 302, 70 μg·kg^−1^). A significant advantage of the method described here is the easier sample preparation and the fast chromatographic analysis. Thus, the current method is substantially more cost-effective and allowed the simultaneous analysis of 18 more pesticides.

Flumethrin has been authorised as an acaricide in Switzerland since 1991 and to date. It was detected in 51% of the analysed samples (> *LOD*) mostly low levels. In 7% of the analysed samples, flumethrin could be quantified (> *LOQ*). The residue levels (mean < *LOQ* and maximum 111 μg·kg^−1^) were below the levels reported for German (mean 160 μg·kg^−1^, maximum 10,900 μg·kg^−1^, Shimshoni et al. 2019), Italian (mean 40 μg·kg^−1^, maximum 110 μg·kg^−1^, Perugini et al. 2018) or Spanish wax (mean 91 μg·kg^−1^, maximum 170 μg·kg^−1^, Calatayud-Vernich et al. 2017). This is most likely because Swiss beekeepers rarely used products containing flumethrin (Bogdanov et al. 2002).

Even though amitraz-containing products have never been authorised in Switzerland for beekeeping, 49% of the foundation batches contained DMF, the breakdown product of amitraz, although at low concentrations. This might be due to the illegal use of amitraz-containing products or due to the import of wax from neighbouring countries, where such products are authorised for mite control (D’Ascenzi et al. 2019).

The insecticides frequently detected in our analysed samples (85–100%) were chlorfenvinphos, chlorpyrifos, permethrin and propoxur. Plant protection products containing chlorfenvinphos were authorised in Switzerland until 2011, but nowadays no such product is authorised for use in beekeeping or as plant protection. The mean and maximal values of chlorfenvinphos (19; 185 μg·kg^−1^) in this study were below the values reported for neighbouring countries, Italy (60; 630 μg·kg^−1^, Perugini et al. 2018) and Germany (200; 6400 μg·kg^−1^, Shimshoni et al. 2019). Chlorfenvinphos has been reported at mean and maximal values of 1491 and 5285 μg·kg^−1^ in Spanish wax foundations, suggesting its unauthorised use as an acaricide in beekeeping (Calatayud-Vernich et al. 2017). Mean and maximal values of chlorpyrifos (< *LOQ*; 34 μg·kg^−1^) in this study were lower than the values reported for German wax (70; 1800 μg·kg^−1^, Shimshoni et al. 2019) or North American wax (24; 890 μg·kg^−1^, Mullin et al. 2010). In Switzerland, the approval for plant protection products containing chlorpyrifos was revoked in July 2020 (de Baan et al. 2020). Thus, levels in wax should decrease over the next years. Permethrin and propoxur are not authorised for use as plant protection in Switzerland. While permethrin is still authorised as a biocide for the protection of wood, propoxur is no longer authorised as a biocide in Switzerland (FOEN, FOPH and SECO 2021). The mean and maximal levels (permethrin mean < *LOQ*, maximum 250 μg·kg^−1^; propoxur mean < *LOQ*, maximum 9 μg·kg^−1^) are below the recently reported residue levels in German wax (mean 170; maximum 2400 μg·kg^−1^ and mean 10; maximum 30 μg·kg^−1^, respectively, Shimshoni et al. 2019). Levels of permethrin were also higher in North American wax (mean 210; maximum 372 μg·kg^−1^; Mullin et al. 2010). Especially chlorpyrifos and permethrin are highly toxic to adult bees through oral or contact exposure (contact LD_50_: 0.01 µg·bee^−1^; oral LD_50_ 0.25 µg·bee^−1^ for chlorpyrifos (Stoner and Eitzer 2013) and contact LD_50_ 0.06 µg·bee^−1^; oral LD_50_ 0.13 µg·bee^−1^ for permethrin (Sanchez-Bayo and Goka 2014), respectively). Larvae tolerate chlorpyrifos mildly better on oral exposure than adult bees (LD_50_ 0.46 µg·larva^−1^, Dai et al. 2017). So far, the exposure route through beeswax is not well studied and it is not clear what concentration level in beeswax leads to increased mortality.

Furthermore, the tested samples contained the fungicides azoxystrobin and boscalid, which are widely used in agriculture to protect cultures, such as berry, stone fruits, vegetables and rape (boscalid). Both are currently authorised for use in Switzerland as plant protection products (FOAG 2021).

Several products containing (E)-fenpyroximate as an active ingredient are currently authorised for use in Switzerland (FOAG 2021). (E)-fenpyroximate is an acaricide used, for example, for the protection of grapes, apples, pears and beans (European Food Safety Authority 2013). It was found in 92% of the tested foundation samples at low levels (mean and maximum concentrations of 4 μg·kg^−1^ and 50 μg·kg^−1^, respectively).

Piperonyl butoxide was found in all the analysed samples, ranging from 6 to 1555 μg·kg^−1^. Similar levels were measured in Italian beeswax (Perugini et al. 2018), while mean values of beeswax from Belgium or Germany were four times lower (El Agrebi et al. 2020; Shimshoni et al. 2019). This substance on its own has low toxicity to mammals (Tozzi 1999), birds and bees (Cross et al. 2017). Instead, it supports the effect of pyrethrum and pyrethroid insecticides, serving as a synergist (Tozzi 1999). Its effect is the prevention of the oxidative enzymatic breakdown of insecticides, mostly by inhibition of cytochrome P450 (Hodgson and Levi 1999). Consequently, the pyrethroid’s toxicity is increased, or the same toxicity is achieved at a lower insecticide concentration (Wickham 1999).

DEET was also detected in all the analysed samples, with a mean concentration of 120 μg·kg^−1^ and a maximum value of 585 μg·kg^−1^ at similar levels reported for Germany (maximum 1300 μg·kg^−1^, Wallner 2014) or Belgium (mean 102 μg·kg^−1^; maximum 707 μg·kg^−1^, El Agrebi et al. 2020). Several years ago, this active ingredient was used as a repellent in beekeeping (Wallner 2014). In the meantime, the formulation of such products has been changed and DEET-containing products for beekeeping are no longer on the market. However, DEET is still used as an insect repellent (Ditzen et al. 2008), e. g. in mosquito sprays. Despite its popularity, the mechanism of toxicity in insects, mammals and humans, as well as the effect on the target olfactory system, is still controversial (Corbel et al. 2009). Indeed, an experiment conducted by Corbel et al. (2009) found that DEET has the potential to inhibit cholinesterase on its own and increase the potency of carbamates, a class of insecticides that block acetylcholinesterase.

## Conclusion

The acaricides coumaphos and *tau*-fluvalinate were the most important residues in Swiss beeswax, followed by the synergist piperonyl butoxide. The residue levels of these two beekeeping-associated acaricides were comparable to the levels reported for wax of the neighbouring countries, while residue levels of piperonyl butoxide were similar to levels in Italian wax, but above the levels measured in wax from Belgium or Germany. On the other hand, the levels of the acaricides flumethrin and DMF, as well as the levels of the insecticides chlorfenvinphos, chlorpyrifos, permethrin and propoxur, were below the levels reported in studies on European wax.

The coumaphos residue levels in Swiss beeswax most likely do not cause lethal brood effects, since the maximal coumaphos level in Swiss wax was seven times below a level that increased brood mortality in an in vitro assay. Additional studies are needed to test brood mortality related to residue levels of a variety of pesticides in beeswax.

## Data Availability

All data generated during this study are included in this article.
